# Combination treatment with trastuzumab and crizotinib in metastatic gastric cancer harboring Her-2 amplification and c-MET amplification: A case report

**DOI:** 10.1097/MD.0000000000027017

**Published:** 2021-09-10

**Authors:** Mingsheng Liu

**Affiliations:** Department of Anus and Intestine Surgery, The Quzhou Affiliated Hospital of Wenzhou Medical University, Quzhou People's Hospital, Quzhou, Zhejiang, China.

**Keywords:** c-MET amplification, crizotinib, gastric cancer, Her-2 amplification, trastuzumab

## Abstract

**Rationale:**

Metastatic gastric cancer patients with poor eastern cooperative oncology group performance status (PS) (≥3) were lack of effective anti-tumor strategies. They always lived with poor PS, severe and multiple symptoms, and usually resulted in extremely limited survival time. Herein, we reported a patient diagnosed with gastric cancer metastasized to multiple bones, along with lymphangitis carcinomatosa in lungs, harboring Her-2 and c-MET amplification with poor PS, positively responded to combinational therapy with trastuzumab and crizotinib.

**Patient concerns:**

The patient complained of persistent cough and fatigue for 2 months, otherwise, she denied smoking, alcohol history, or any other medical or family history.

**Diagnosis:**

With the biopsy results from gastroscopy, as well as computer tomography for chest and abdomen, the patient was diagnosed as gastric adenocarcinoma, with metastasis on lungs, left adrenal gland, retroperitoneal lymph nodes, and multiple bones.

**Interventions:**

Because of the poor PS (PS = 3), as well as Her-2 and c-MET amplification, the patient received combination treatment with trastuzumab and crizotinib as salvage strategy.

**Outcomes:**

After 2 months’ exposure of trastuzumab and crizotinib, symptoms including persistent cough, and chest distress were alleviated significantly. Simultaneously, chest computer tomography showed significant dissipation of lymphangitis carcinomatosa, as well as apparent reduction of pleural effusion. No adverse reactions including nausea, vomiting, diarrhea, or hypertension was observed during the following 2 months.

**Lessons:**

The present case suggested that combinational therapy with trastuzumab and crizotinib might be effective in metastatic gastric cancer patients harboring Her-2 and c-MET amplification, even with a poor PS. It was also implied that gene sequencing might be valuable, especially in patients with limited treatment strategies.

## 1 Introduction

Metastatic gastric cancer has been deemed a cancer with high heterogeneity that rapidly progresses, and is known for its poor prognosis, especially in patients with bone metastatic disease.^[1]^ The median overall survival of gastric cancer patients with bone metastasis was less than 6 months.^[2]^ Metastatic gastric cancer patients with poor eastern cooperative oncology group performance status (PS) (≥3) were especially positioned as contraindication for anti-tumor therapy, and usually resulted in extremely limited survival time. They always merely received supportive care, which usually resulted in unsatisfactory outcomes. Herein, we report on a patient diagnosed with gastric cancer that had metastasized to multiple bones, along with lymphangitis carcinomatosa in the lungs, harboring Her-2 amplification and c-MET amplification with poor PS (PS = 3) that positively responded to a combinational therapy of trastuzumab and crizotinib. After being exposed to the combined treatment of trastuzumab and crizotinib, she achieved a partial response, saw an improvement in quality of life, as well as a significant relief in lymphangitis carcinomatosa.

## 2 Case presentation

**Figure 1:**
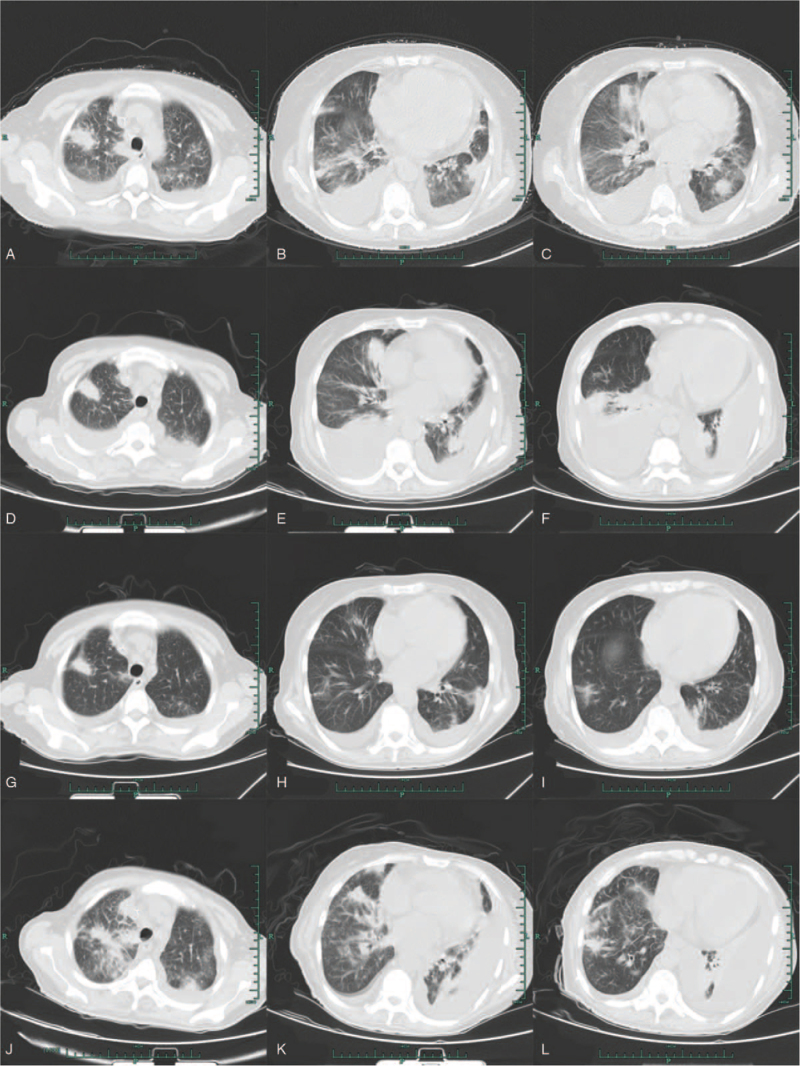
Chest CT scanning showing the metastatic lymphangitis carcinomatosa in lungs. A–C. April 12, 2020; D–F. May 12, 2020; G–I. May 19, 2020; J–L. July 9, 2020. CT = computer tomography.

**Figure 2:**
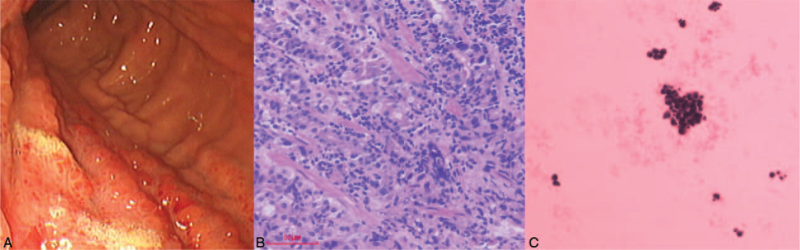
Endoscopy outcomes (A), and histological findings with hematoxylin and eosin-stained biopsy specimen from gastroscopy (B, ×40), and pleural effusion (C, ×40).

**Figure 3:**
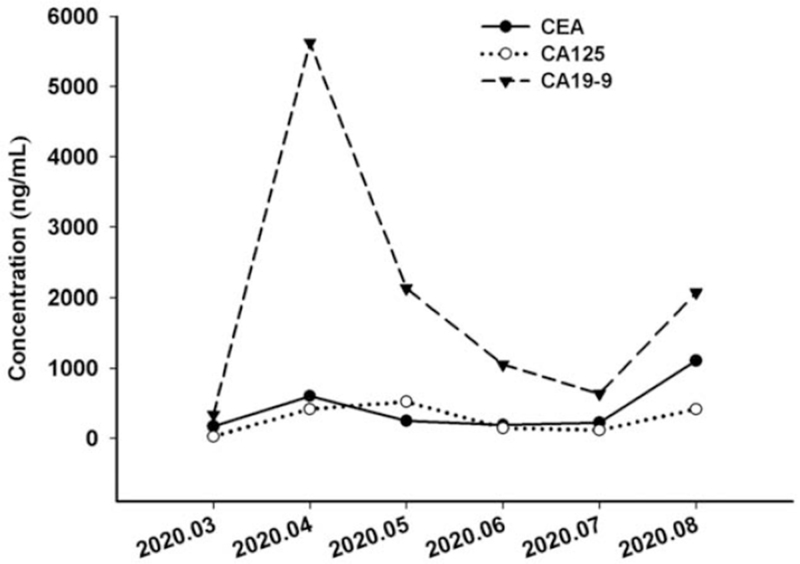
The variations of tumor markers including CEA, CA125, and CA19-9 during the whole treatment.

A 59-year-old woman was admitted to our institution on April 9, 2020 with a persistent cough and fatigue that had lasted for 2 months. She denied having a history of smoking or drinking, as well as any other medical or family history. Results of chest computer tomography (CT) suggested diffuse infiltration and interstitial edema on both of the lungs (Fig. 1A–C). A subsequent abdomen CT revealed a suspicious mass on her left adrenal gland, enlarged retroperitoneal lymph nodes, and multiple destruction of bones, including lumbar vertebrae, sacrum, and ilium. Because of the abnormally enlarged retroperitoneal lymph nodes, endoscope examinations, including gastroscopy and enteroscopy were scheduled. The gastroscopy findings suggested ulcerations in the lesser curvature of the stomach, which was finally identified as gastric adenocarcinoma with a poor differentiation degree (Fig. 2A–B). Immunohistochemistry outcomes were presented as: CDX-2 (positive), CEA (positive), Her-2 (2+), Ki-67 (60%), WT-1 (negative), CK5/6 (negative), CK7 (positive), β-catenin (positive), and CA199 (positive). Her-2 amplification positive was further identified with fluorescence in-situ hybridization. Based on these tests, the patient was ultimately diagnosed with gastric adenocarcinoma, with metastasis in the lungs, left adrenal gland, retroperitoneal lymph nodes, and multiple bones, which was identified as stage IV (T3N2M1 by the American Joint Committee on Cancer, 8th version).^[3]^ Because of the poor PS (PS = 3), we did not administer any cytotoxic drugs as front line treatment. Instead, monotherapy with trastuzumab at a dosage of 4 mg/kg the first week, followed by 2 mg/kg weekly was prescribed as a salvage treatment strategy. Simultaneously, next generation sequencing, including 50 tumor-related genes that utilized tissue and plasma samples was conducted to search for potential targets. After 2 weeks on trastuzumab and palliative care, the fatigue and poor appetite improved. However, the persistent cough and chest distress became even further aggravated. Repeated chest CT on May 12, 2020 revealed increased pleural effusion, and advanced to deteriorative interstitial edema in both of the lungs (Fig. 1D–F). Malignant pleural effusion was identified based on the findings from the malignant cells from pleural effusion (Fig. 2C). Although with pleural effusion drainage of 500 to 1000 mL daily, the cough or chest distress was not effectively relieved. Cough medications, including compound methoxyphenamine capsules, codeine phosphate, or montelukast showed no efficacy, On May 11, delayed next generation sequencing reported a 9.2-fold c-MET amplification in plasma levels and 4.4-fold in tumor tissue levels. As a salvage treatment, oral crizotinib was additionally administrated at a dosage of 250 mg twice a day from May 12, 2020. Simultaneously, weekly trastuzumab was still managed as before. Surprisingly, after only 2 days of the combination treatment, symptoms, including the persistent cough, and chest distress, which may have been caused by the lymphangitis carcinomatosa in lungs, were alleviated to a large degree. One week later, her PS improved from 4 to 2. We conducted an extra chest CT on May 19, 2020 to investigate the metastatic disease in lungs, although it was only 1 week after the previous chest CT was conducted. As a result, the results from the chest CT showed that lymphangitis carcinomatosa had dissipated by a large degree, not to mention an apparent reduction in pleural effusion, which should not have seen improvement from mere punctures of pleural effusions (Fig. 1 G–I). Based on the superior efficacy of the combinational regimen and improvement in PS, we recommended standard chemotherapeutic regimens, including FOLFOX (Oxaliplatin plus fluorouracil) or SOX (Oxaliplatin plus S-1) as sequential treatment. However, the patient refused any cytotoxic drugs, and discharged then. At that point she had completed 2 months of the combined trastuzumab and crizotinib treatment at the clinic. The best response status was evaluated as a partial response, according to the criteria of the Response Evaluation Criteria in Solid Tumors (version 1.1).^[4]^ No adverse events, including nausea, vomiting, diarrhea, or hypertension were observed during the following 2 months, according to the criteria of National Cancer Institute Common Terminology Criteria for Adverse Events version 4.0.^[5]^ On July 9, 2020, the patient was admitted to our hospital again because of an aggravated cough. A chest CT revealed the exacerbation of deteriorative interstitial edema (Fig. 1J–L). However, other lesions, including the left adrenal gland, enlarged retroperitoneal lymph nodes, and multiple destruction of bone were evaluated as a stable disease. Based on the image results, the patient was evaluated as having a progressive disease then. Although with active and supportive care, including draining pleural effusion, the symptoms from the chest distress did not improve. The patient died of respiratory failure in August 2020. The variations in tumor markers, including CEA, CA125, and CA19-9 during the whole treatment process were presented in Figure 3.

## 3 Discussion

The present case study reported on a patient diagnosed with metastatic gastric cancer, along with lymphangitis carcinomatosa in the lungs, harboring Her-2 and c-MET amplification with poor PS (PS = 3), positively responded to combination treatment of trastuzumab and crizotinib. With the combinational targeted therapy, the patient achieved a partial response, an improvement in PS, as well as significant relief from lymphangitis carcinomatosa. The present case suggested that combinational therapy with trastuzumab and crizotinib might be effective in metastatic gastric cancer patients harboring Her-2 and c-MET amplification, even with poor PS. It also implied that gene sequencing might be valuable, especially in patients with limited treatment strategies.

In the last decade, Her-2, as a member of the Her-family of receptors, was recognized as a key driver of tumorigenesis, as well as an important therapeutic target in gastric cancer, with studies showing amplification or over-expression in 7% to 34% of tumors.^[6]^ It is associated with tumor cell proliferation, migration, survival, and angiogenesis and usually correlates with a poor prognosis and a more aggressive disease.^[7]^ Trastuzumab, a monoclonal antibody against the Her-2 receptor, inhibits tumor growth primarily by preventing Her-2 homodimerization and blocking Her-2 downstream signaling, inhibiting Her-2 extracellular amino-terminal domain shedding, and inducting antibody-dependent cellular cytotoxicity.^[8]^ Trastuzumab in combination with chemotherapy has already been recommended as a standard option for patients with Her-2-positive advanced or metastatic gastric or gastro-oesophageal junction cancer based on the positive results from the trial ToGA.^[9]^ However, the ToGA only enrolled patients with good PS (PS ≤ 2), which restricted the applicable population in clinical practices. There were few small-scale studies that investigated the efficacy and safety profile of anti-Her-2 treatment in patients with poor PS. A case study reported that a patient with Her-2 over-expressing metastatic gastric cancer that was accompanied by bone marrow involvement and severe thrombocytopenia, positively responded to trastuzumab monotherapy.^[10]^ The progression-free survival time for that patient was 5 months. However, in the present case, administering monotherapy with trastuzumab failed to relieve chest distress symptoms or improve lymphangitis carcinomatosa in lungs, which suggested that anti-Her treatment might not be effective for metastatic tumor cell subgroups in lungs. The disease in the lungs saw significant improvement until the introduction of crizotinib, a prototype type-I MET inhibitor with a U-shaped binding mode at the ATP binding site, which showed tremendous promise in treating non-small cell lung cancer and gastric cancer patients harboring MET amplification.^[11–13]^ Based on that, it was posited that the tumor subgroups in the lungs might be primarily driven by c-Met amplification, rather than Her-2 amplification. However, without drive gene tests for tumor cells in lungs, the heterogeneity of drive the genes among metastatic organs could not be absolutely identified in the current case. In addition, there was another case that reported the c-MET amplification as the drive gene in a gastric cancer patient.^[14]^ A former publication reported a case of c-MET amplification in advanced gastric cancer with liver metastasis. With 2 months having crizotinib administered, liver lesions were completely in remission, and progression-free survival lasted for up to 20 months. Moreover, an acquired sensitive mutation of the c-MET amplification could also be inhibited in patients with non-small cell lung cancers according to a recent piece of literature.^[15]^ Hence, it was implied that c-MET amplification could also be a candidate drive gene in patients with gastric cancer in a particular situation. In the current case, we considered that the salvage regimen of trastuzumab and crizotinib provided an opportunity for sequential systematic treatment by improving the PS, and relieving symptoms. If the patient received further cytotoxic treatment at the point of better PS, the survival outcome might have improved. In addition, it was also suggested that gene sequencing might be valuable, especially in patients with limited treatment strategies.

In summary, we presented a case diagnosed with metastatic gastric cancer, harboring Her-2 and c-MET amplification with poor PS (PS ≥ 3) that positively responded to the combination treatment of trastuzumab and crizotinib, which suggested that the combinational targeted regimen of trastuzumab and crizotinib might be effective in metastatic gastric cancer patients harboring Her-2 and c-MET amplification, even with a poor PS.

## Acknowledgments

The authors thank the patient for her participation and her relative's agreement to publication of the report.

## Author contributions

**Conceptualization:** Mingsheng Liu.

**Data curation:** Mingsheng Liu.

**Formal analysis:** Mingsheng Liu.

**Investigation:** Mingsheng Liu.

**Methodology:** Mingsheng Liu.

**Software:** Mingsheng Liu.

**Writing – original draft:** Mingsheng Liu.

**Writing – review & editing:** Mingsheng Liu.
